# Supplementary material for: Intraoperative calculus or hemorrhage in transurethral seminal vesiculoscopy as a risk factor for recurrent hemospermia
Source: PLoS One. 2022 Jul 5;17(7):e0268314. doi: 10.1371/journal.pone.0268314 (PMC9255724; doi:10.1371/journal.pone.0268314)
Supplement: Attachment — Submitted filename: Response to Reviewers.docx [file pone.0268314.s002.docx]

Wen-Wei Sung, M.D., Ph.D.

Academic Editor

PLOS ONE

[Submission Date: April 26, 2022]

Dear Dr. Sung:

Thank you for offering us the opportunity to submit a revised draft of our manuscript entitled, Intraoperative calculus or hemorrhage in transurethral seminal vesiculoscopy as a risk factor for recurrent hemospermia to PLOS ONE. We also appreciate the time and effort you and each of the reviewers have dedicated to providing insightful feedback on ways to better strengthen our paper. Thus, it is with great pleasure that we resubmit our article for further consideration. We have incorporated the necessary changes which reflect the detailed suggestions you have graciously provided. We also hope that our edits and provided responses satisfactorily address all the issues and concerns you and the reviewers have noted.

To facilitate your review of our revisions, the following is a point-by-point response to the questions and comments delivered in your letter dated 12^th^ May 2022 and 2^nd^ June 2022.

**REVIEWER 1 COMMENTS:**

1. Minor structural or grammatical errors need to be taken into account, e.g., “Transrectal Ultrasound (TRUS), Computed Tomography (CT), and Magnetic Resonance Imaging (MRI)” in line 29 should use small letters instead of capital letters. Also, an inconsistent style has been used to present the number of patients, e.g., “Forty-eight (48) patients” in line 74 versus “22 non-recurrent patients” in line 77.
   - Thank you for your suggestions. We have corrected our manuscript as per your comments.
2. The citations at the end of the sentence should always be put before the full stop, e.g., Hemospermia, hematospermia, or haematospermia is defined as blood appearance in the 26 semen [1].
   - Thank you for your suggestion. We have corrected our manuscript as per your comments.
3. Residual confounders such as lifestyle and the influence of comedications were not considered in the current study, which should be addressed in the limitations section.
   - You have raised an important issue. We have rewritten the Limitations and future prospects section of the paper (p. 13-14, lines 192-195) to be more in line with your comments.

**REVIEWER 2 COMMENTS:**

1. Recurrent hematospermia is an important problem in clinical practice. The authors include patients with persistent hemospermia and intractable hemospermia, with symptoms persisting over 3 months regardless of medical treatment. Is there any guideline or consensus define the definition of intractable hemospermia.
   - You have raised an important question. We have rewritten the Introduction (p. 3, lines 32-36) to be more in line with your comments.
2. The succussful rate of transurethral seminal vesiculoscopy is very high in this study. Are all patients received complete TUSV? Is there any patient have receive only one side succussful TUSV
   - You have raised two important questions. We have rewritten the Success rate section found under the Discussion heading (p. 12-13, lines 171-178) to be more in line with your comments.
3. Intraoperative manifestations, such as Calculus, hemorrhage, mucosal lesion, were recorded. Please provide how many patient have mucosal lesion and the biopsy result.
   - Thank you for your suggestion. We have added the result in Table 2(p. 8) and in the Intraoperative findings section (p. 8, lines 95-98). Raw data was added in S1 File.

**ETHICS STATEMENT**

1. Please provide additional details regarding participant consent. In the ethics statement in the Methods and online submission information, please ensure that you have specified (1) whether consent was informed and (2) what type you obtained (for instance, written or verbal, and if verbal, how it was documented and witnessed). If your study included minors, state whether you obtained consent from parents or guardians. If the need for consent was waived by the ethics committee, please include this information.
   If you are reporting a retrospective study of medical records or archived samples, please ensure that you have discussed whether all data were fully anonymized before you accessed them and/or whether the IRB or ethics committee waived the requirement for informed consent. If patients provided informed written consent to have data from their medical records used in research, please include this information
   - Thank you for your suggestion. We have rewritten the Patients section found under the Materials and Methods heading (p. 4, lines 55-56) to be more in line with your comment.

**Concluding remarks**: Again, thank you for giving us the opportunity to strengthen our manuscript by following your valuable comments and queries. We have worked hard to incorporate your feedback and trust that these revisions will help towards your acceptance of our submission.

Sincerely,

Chen, Chuan‐Shu, M.D.

Division of Urology, Department of Surgery, Taichung Veterans General Hospital, Taichung, Taiwan

E-mail: r2060d@gmail.com
